# In Vitro 3D Modeling of Neurodegenerative Diseases

**DOI:** 10.3390/bioengineering10010093

**Published:** 2023-01-10

**Authors:** Aurélie Louit, Todd Galbraith, François Berthod

**Affiliations:** 1 LOEX, Centre de Recherche du CHU de Québec-Université Laval, Québec City, QC G1J 1Z4, Canada; 2 Department of Surgery, Faculty of Medicine, Université Laval, Québec City, QC G1J 1Z4, Canada

**Keywords:** scaffolds, bioprinting, spheroids, organoids, microfluidic, organ on chip

## Abstract

The study of neurodegenerative diseases (such as Alzheimer’s disease, Parkinson’s disease, Huntington’s disease, or amyotrophic lateral sclerosis) is very complex due to the difficulty in investigating the cellular dynamics within nervous tissue. Despite numerous advances in the in vivo study of these diseases, the use of in vitro analyses is proving to be a valuable tool to better understand the mechanisms implicated in these diseases. Although neural cells remain difficult to obtain from patient tissues, access to induced multipotent stem cell production now makes it possible to generate virtually all neural cells involved in these diseases (from neurons to glial cells). Many original 3D culture model approaches are currently being developed (using these different cell types together) to closely mimic degenerative nervous tissue environments. The aim of these approaches is to allow an interaction between glial cells and neurons, which reproduces pathophysiological reality by co-culturing them in structures that recapitulate embryonic development or facilitate axonal migration, local molecule exchange, and myelination (to name a few). This review details the advantages and disadvantages of techniques using scaffolds, spheroids, organoids, 3D bioprinting, microfluidic systems, and organ-on-a-chip strategies to model neurodegenerative diseases.

## 1. Introduction

Neurodegenerative diseases encompass a wide range of clinically and pathologically diverse pathologies affecting the central nervous system. These diseases can induce progressive cognitive impairments affecting memory loss or affect motor functions (or both). The most common neurodegenerative diseases are Alzheimer’s disease (AD), Parkinson’s disease (PD), Huntington’s disease (HD), and amyotrophic lateral sclerosis (ALS) (Table 1) [1,2,3]. The study of pathologies affecting the brain or the spinal cord are particularly challenging due to the complexity of these tissues and the difficulty in accessing them. The characterization of the impact of these pathologies on the nervous tissues (such as protein aggregation) can be carried out on post-mortem tissues, however, they do not allow for a clear understanding of the mechanism of the pathology.

In vivo disease studies have led to great discoveries, and even today animal models are still widely used (especially for pre-clinical studies). However, technical and ethical limits appear, as well as a doubt concerning the reliability of transposing these results to humans [4,5].

In vitro studies oriented on the cells involved in neurodegenerative diseases have enabled researchers to focus on close interactions between cells (such as neurons, astrocytes, microglia, and oligodendrocytes), to better understand the disease mechanism at the cellular and molecular levels. In addition, these in vitro culture systems allow researchers to co-culture together (in various combinations) diseased or healthy cells to more accurately determine which cell type causes or aggravates the pathology [6].

Many studies have turned to in vitro two-dimensional (2D) monolayer cell culture and, more recently, studies using three-dimensional (3D) cell culture models. However, since neural cells are difficult to extract from patients, animal cells have become commonplace in neurodegenerative disease studies, but with major disadvantages, such as a difficulty to model age-related diseases. There is, therefore, growing interest in patient-derived induced pluripotent stem cells (iPSC), which have the potential to differentiate into various neural cells while preserving patients’ genetic backgrounds [7,8]. Two-dimensional cell culture has allowed researchers to answer many questions, such as cell growth or cell behavior in response to stress (drugs, pathogens, etc.), but shows shortcomings in reproducing the physiological environment due to a lack of cell–extracellular matrix (ECM) and cell–cell interactions [9,10]. Thanks to a more complex and adjustable physiological environment that combines together ECM with various cell types, the use of 3D cell culture models is growing, and brings additional responses to in vivo studies. A variety of neurodegenerative disease models have been developed (using a scaffold-based approach, spheroid, or organoid techniques) and have demonstrated control over cell organization in some models, and flexibility regarding ECM material selection for others [6,11,12,13,14,15]. However, a deficiency in both nutrient/oxygen supply and waste elimination were often some of the limitations pointed out when using these engineer-based models. In recent years, more automated and reproducible tools, such as 3D bioprinting, microfluidic systems, and organs-on-chips have emerged and could overcome issues such as inadequate oxygenation/nutrition, precise spatio–temporal control, etc. [16,17,18].

In this review, we discuss the benefits and drawbacks of in vitro cell culture models compared to in vivo models. We continue with the advantages and differences that 3D cell culture can bring compared to 2D cell culture. Finally, we provide an overview of different technologies that can be used for 3D modeling.

## 2. In Vivo Models

In the context of neurodegenerative diseases, in vivo models allow a certain complexity and help greatly in the understanding of brain functions. There are many in vivo models recapitulating neurodegenerative diseases (such as AD, PD, ALS, etc.) and are constantly evolving with humanized models. Windrem and Benraiss’s studies have shown that mouse glia can be replaced by glial progenitors differentiated from patient-derived iPSCs in immunodeficient mice to study schizophrenia or Huntington disease [19,20]. Animal models are widely used for preclinical studies such as in ALS, where they have generated endless data about motor neuron degeneration [21]. However, there are several issues to consider when using animal models. Indeed, in vivo models are different from human ones (in their structure, their function, and their metabolism). Consequently, it is difficult to study key molecular and cellular mechanisms.

The pathological hallmarks of AD (responsible for cognitive impairments) are the formation of β-amyloid senile plaques and neurofibrillary tangles of hyperphosphorylated tau protein. Various transgenic mouse models were developed, such as the PDAPP or Tg2576 mice overexpressing a mutated patient-derived protein, and were successful to recapitulate cognitive impairment and β-amyloid plaque accumulation. However, they did not reproduce the tau pathology as well as the extensive neuronal loss seen in patients (at least in part) because they do not consider all the epigenetic factors observed in humans [22,23,24]. In addition, diseases may not affect animals and humans in the same way, and, conversely, animals and humans can respond differently to the illness [23,24].

Modeling PD in vivo can be achieved easily though the injection of the neurotoxins MPTP or 6-hydroxydopamine in the striatum of rodents or apes, but it will only recapitulate the motor impairment of PD [25]. To more accurately mimic the disease, mice knock-out for Parkin or Pink1 were tested, as well as some transgenic mouse models to reflect the synucleinopathy detected in dopaminergic neurons in humans [26]. However, they only resulted in partial dopaminergic neuron degeneration [26,27].

HD that induces deadly motor and cognitive dysfunction is caused by an expansion of CAG repeats in the huntingtin gene. Some transgenic mouse models overexpressing this human CAG expansion were developed (such as the R6/2 mouse model). However, these models only partly reproduce the cognitive and motor deficits, as well as neuron loss, observed in HD [28].

Another drawback of these models is that they only allow for the study of certain mutations, but they do not enable studying sporadic conditions which represent 90% ALS cases [29,30]. In this deadly disease, which induces motor neuron degeneration, a mouse model overexpressing the human mutated SOD1 protein, recapitulating motor disfunction and death, has been used extensively over the last thirty years. However, the mechanism leading to motor neuron death still remains unclear [15].

Although in vivo models are extremely useful in the preclinical phase or in new drugs testing, they are expensive, time-consuming, and have a low success rate. Indeed, less than half of the drugs tested in phases II and III will be translated to the clinic [4,5]. In addition to ethical issues that may be related to the use of animals, compliance to the “3Rs rule” (replace, reduce, refine) will lead to additional limitations [31]. Setting up new in vitro models to better understand disease mechanisms and studying new drugs is a growing need.

## 3. In Vitro Models

In vitro models represent a valuable approach to modeling diseases and cellular mechanisms. Interestingly, they allow for the independent study of cell interactions (one cellular or molecular mechanism at a time). Whether in 2D or 3D, one of the recurring problems is the origin of the cells used in these models. Cells are often extracted from animals, hence, in vitro modeling faces the same disadvantages which were previously listed for in vivo models. Cells can be extracted from an embryonic or post-natal brain and glial cells can be expanded quickly. However, their use is restricted to availability and relevance of the disease mouse model, not to mention that it is much more complicated to replicate age-related diseases from fetal neurons. Other limitations due to cells or culture conditions are well-reviewed in Horvath’s paper [32]. Recently, interest is growing towards iPSCs, which can self-renew and differentiate into cells of all three germ layers [33]. iPSCs are pluripotent stem cells obtained by reprogramming somatic cells using Yamanaka factors through methods that do not affect the cell genome integrity [7]. The iPSC utilization in in vitro models allows researchers to avoid ethical problems related to the use of embryonic stem cells. Furthermore, using iPSCs could enable a reduction of the use of animals in research. They can help to model diseases and serve as a screening platform [34,35,36]. These cells could have an advantage over cells extracted from animals because they have the capability of long-term culture, come from human diseased tissues, and can preserve patient characteristics [8]. Moreover, iPSCs could be very useful for personalized medicine, as they might preserve, at least in part, the disease phenotype and genetic inheritance. They also allow for the development of polygenetic models, which is more laborious to perform with animals. Despite their advantages, iPSCs suffer from some limitations, such as the lack of standardized protocols, which greatly impacts reproducibility. Differences between cell lines can occur because of the genetic background of the cells, and necessitate expensive production of isogenic iPSCs to serve as controls [37]. Another inconvenience that affects the modeling of neurodegenerative diseases (often known as late-onset diseases, i.e., AD or ALS) is the loss of epigenetic factors and age-related markers during iPSC reprogramming [38,39,40,41]. Fortunately, some strategies, such as using cells with fewer genetic mutations, safer reprogramming protocols, or direct reprogramming, may help to overcome these limitations [42,43,44].

### 3.1. 2D Cell Culture

**Figure 1:**
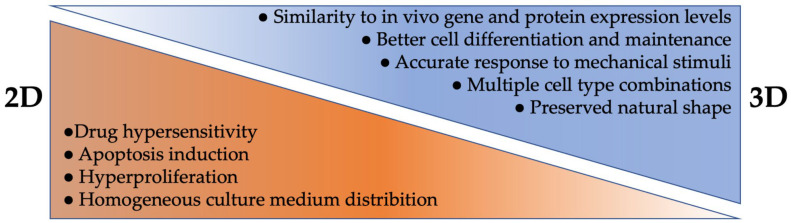
Main characteristics of in vitro cell culture methods performed in 2D versus 3D conditions.

For many years, 2D cultures (usually as a cell monolayer on a rigid substrate) have been used to study cellular interactions and responses to chemical, physical, or mechanical stimuli, etc. Actually, 2D cell culture is a fast and simplified approach compared to the 3D one. It is less expensive, and easier to manipulate, reproduce, and analyze results [45]. However, although 2D culture has significantly enhanced our understanding of cellular growth and behavior, some limitations are still emerging. Tissues and organs are made of many different cell types interacting together in a 3D environment. Thus, a 2D culture does not allow for the reproduction of this high level of complexity [9,10,46,47]. Culturing cells in a monolayer may induce morphology changes, can affect their functions, their organization, and their secretome or signaling pathways (Figure 1) [48,49,50]. In addition, 2D culture also modifies cell division and triggers some phenotypic loss, a polarity change which can enhance apoptosis, and/or increase proliferation [51,52,53]. Another drawback of 2D culture is that monolayer culture may alter gene and protein expression levels, as well as extracellular vesicle release, when compared to a 3D environment [52,54,55,56]. Furthermore, since the culture medium is homogeneously distributed, cells initially have unrestricted access to nutrients, oxygen, and growth factors, but over time, the levels decrease in amounts until the next culture medium renewal. This same pattern is repeated for waste removal, which is in contrast with what happens in vivo [50]. Additionally, drugs tested on cells grown in 2D often have ineffective therapeutic potential due to their abnormal morphology [57,58,59]. When cells are grown on flat surfaces, their spatial arrangement is different, which causes cell surface receptors to be more exposed to drugs than when embedded within a 3D tissue [57,58,59]. In 2D culture, cells have a tendency to dedifferentiate to a fibroblast-like state. As a result, cells proliferate more and are much more affected by a drug’s activity, which often requires proliferating cells to be effective [59,60,61]. An increased pH observed in 2D can also affect the effectiveness of the drugs. Usually, a higher pH is more conducive to an increase in the drug effectiveness [62,63]. For all these reasons, many drugs tested in 2D are not potent or do not reproduce the expected effects in later stages of development [64].

### 3.2. 3D Cell Culture

To study tissue or cellular mechanisms occurring in vivo, in vitro 3D culture models are increasingly used (Figure 1). First of all, a great advantage in 3D models is the presence and involvement of an enriched ECM, creating models with a closer physiology to the ones found in vivo. In addition to its support function, the presence of ECM exposes junction and adhesion molecules on its surface, which helps in promoting growth factor expression and interactions, both with and between cells [65,66]. ECM can also play a role in the mechanisms of cell spreading, differentiation, migration, and proliferation. Oxygen, nutrient input, and waste clearance are also under ECM control, which is an advantage over a 2D culture that maintains cells in a high concentration nutrient bath with a rapid waste diffusion [39,66]. Another major benefit of 3D models is the possibility to co-culture several different cell types together, allowing for complex cell arrangements and cell interactions. This enables the ability to compare different versions of each model with or without a specific cell type, and to use different design techniques such as bioprinting, organoids, or spheroids, as detailed further below. Three-dimensional models allow researchers to modulate the microenvironment and the environmental physical properties, such as porosity, stiffness, or thickness, according to the needs of the cell type. It has been reported that cell morphology remains stable in a 3D model and thus, this brings another advantage to using 3D cell culture growth [58]. For example, in 3D culture, neurons are more rounded than in 2D, and neurites can spread in all directions (like in vivo) and contact surrounding cells, similar to an in vivo environment [67]. Some limitations found in 2D culture can be overcome in 3D culture, where cell polarization and a faster cell differentiation are often observed [68]. Moreover, by adjusting the culture material used and its physical properties, it is also possible to impact the gene expression to get as close as possible to an in vivo setting [52,69]. Although 3D culture represents a great technological advance with many advantages, some limitations appear depending on the 3D method used, and many challenges remain to be solved. First, 3D culture is more expensive, time consuming, and complex to use than 2D culture. Indeed, depending on the method used, some equipment can be very expensive, requiring bioreactors to maintain certain cell types in culture. In addition, the technologies used to reproduce the microenvironment can also be expensive, and some techniques are difficult to reproduce (e.g., organoids). This may be due to a lack of standardized protocols or because of a batch change in the materials or products used for the culture [43,50]. Imaging 3D culture can be more complicated due to the thickness of the materials or scaffold restrictions. Recent advances in tissue clearing technologies have been developed to counter this limitation [70]. Often, 3D models do not mimic animal models or human physiology enough. The goal should not be to simply co-culture cells, but to create separated physical compartments able to interact with each other to create tissue–tissue interfaces. It is also important to consider the composition of the ECM, as well as the biochemical effects involved in the culture systems [71,72]. It may also be difficult to access some cells, and, therefore, to perform functional or electrophysiological analyses such as a patch clamp. Another drawback in 3D culture is that cells are often not exposed to tension, shear stress, compression forces, or flowing fluid as they are in vivo [71,72]. In addition, the absence of a homogeneous diffusion of the culture medium inside the construct, mimicking vascularization, can often be the cause of a deficit in oxygen and nutrient distribution necessary for the formation, survival, or maturation of several tissues. Depending on the 3D technology used, it is possible to overcome this limitation by applying dynamic flow methods, such as a pressure gradient. Choi and his colleagues employed osmotic pressure to mimic the brain interstitial flow [73]. Even if they are related and seem to have a key role in several neurodegenerative diseases [74,75], the absence of inflammatory cells such as microglia in certain models of neurodegenerative diseases can be noted [47]. Herland et al., developed a 3D model of the human blood–brain barrier in a microfluidic chip by culturing human endothelial cells with human pericytes or astrocytes. With this innovative tool, they were able to study the human neurovascular components in vitro and analyze the physiological contributions of different cell types [76]. Finally, the 3D modeling of certain brain-specific regions remains hard to achieve [47,77]. To continuously improve in vitro 3D models, several teams are taking up various challenges to overcome existing shortcomings and attempt to model neurodegenerative diseases. Some research groups are mixing technologies, such as differentiated cells obtained from iPSCs and microfluidic platforms, to study interactions between cells and the vascular compartment, for example [78,79]. Others are studying interactions between neuronal and non-neuronal cells, such as microglia or astrocytes, which have key roles in the formation and synaptic function involving growth factor secretion [80]. Herein, we describe the different 3D approaches used in vitro with their advantages and disadvantages (summarized in Table 2).

#### 3.2.1. Scaffold-Based Approach

Depending on the chosen material, scaffolds can have various advantages. First, a scaffold-based approach is often highly reproducible and offers control over the ECM organization. Secondly, scaffold-based models can provide the physicochemical properties necessary for cell adhesion, proliferation, differentiation, and survival, while enabling cell interactions with the ECM [81,82,83]. Finally, thanks to their permeability, these models allow the diffusion and exchange of nutrients, growth factors, and oxygen. The size of the samples, as well as their degradation speed, depend on the scaffold used, and can vary up to a few centimeters for the size and between 1 day and several months for the biodegradation follow up. Different categories of materials can be used to develop these models depending on the analysis to be carried out. One possible approach is using animal or human-based scaffolds, which consists of decellularizing a native tissue or organ while maintaining the integrity of the ECM and the active molecules embedded inside. This provides an intact 3D microenvironment for cell growth and development [84,122]. Both natural and synthetic polymers are used in the scaffold-based approach. Each individual polymer presents different advantages, and can bring rigidity, elasticity, resistance, hydrophilicity, low immunoreactivity, and appropriate degradability to a model. Natural materials are sought after for their biocompatibility, whereas synthetic materials are chosen for their (mechanically, chemically) customizable aspects and resistance to biodegradation, which helps avoid risks of scaffold remodeling. To combine the benefits of natural and synthetic polymers, several research teams have created hybrid materials where ECM proteins can be integrated into synthetic materials such as hydrogels. Hydrogels are a network of natural polymers (agarose, collagen, silk, chitosan, gelatin, Matrigel^TM^, etc.), synthetic polymers (PEG, polyvinyl acetate), or both, with high versatility, good hydrophilicity, and low toxicity [6,85]. Since natural polymers do not have strong mechanical properties, they can be combined with synthetic polymers for increased strength. These hybrid hydrogels can have similarities with different CNS tissue characteristics, and can be used to model healthy or pathological neural tissues [11]. In general, hydrogels are still widely used to study neural cell culture, as reviewed in De la Vega and Murphy’s papers [66,123]. Depending to the type of polymer, the scaffold-based approach may have some drawbacks. For example, the composition of the material, its porosity, and its rigidity may modify cell growth or adhesion, and the topography of the cell distribution may modify cell behavior [43,46,85,86]. Even if this approach remains highly reproducible, one should not neglect the variability that can occur between different polymer batches.

#### 3.2.2. Spheroids

**Figure 2:**
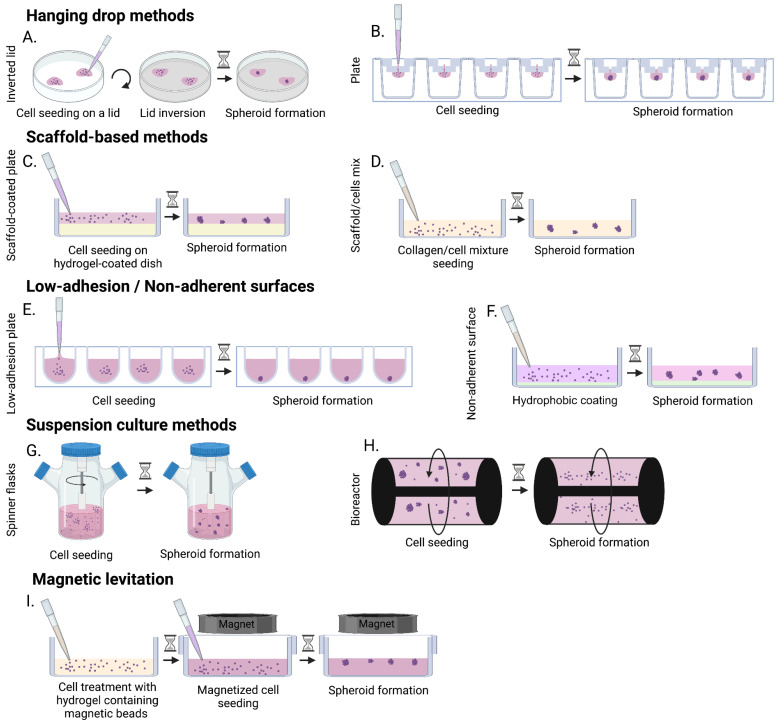
Common techniques used to generate 3D spheroids. Hanging drop methods: Either a suspension of cells is deposited on a petri dish lid which is inverted over a petri dish (**A**), or in a well and passes through a micro-well to form a drop (**B**). Scaffold-based methods: Either cells are seeded on a hydrogel-coated petri dish (**C**) or they are embedded in the hydrogel and seeded in a petri dish (**D**). Low-adhesion/non-adherent surfaces: Cells are seeded in low-adhesion wells to form one spheroid per well (**E**) or on non-adherent surface coated with a hydrophobic substance to prevent cells from spreading (**F**). Suspension culture methods: Cells are placed in spinner flasks (**G**) or in bioreactor (**H**) and put under dynamic culture conditions. Magnetic levitation: Cells are first prepared with magnetic beads and then placed in low-adhesion plates with a magnet on the lid (**I**). Figure produced with Biorender.com.

Spheroids are multicellular aggregates formed in a spontaneous or forced way using low adhesion plates, hanging drop, suspension cultures, or magnetic levitation techniques. These different methods are described below and illustrated in Figure 2 [90]. The self-assembly of spheroids does not require the addition of any biomaterial, and the ECM is only produced by the cells themselves [17,124,125]. Spheroid modeling allows for the creation of an in vitro cell niche similar to in vivo microenvironments. Spheroids have a defined geometry, with a size generally smaller than 1 mm, and can be maintained in culture for up to approximately 2 months [45]. Spheroids are often used to model 3D cancer tumors, but they have also been used for the characterization of nerve tissue (cell diversity, electrophysiology, mechanical rigidity, and ultrastructure) [17,124]. This is a reproducible 3D model with oxygen and nutrient gradients that lead to a heterogeneous cell population with physiologically optimal cell–cell and cell–ECM interactions. However, several studies have shown that a larger spheroid diameter had an impact on the oxygen and nutrient diffusion, and, above a certain size, the authors observed the presence of hypoxic and necrotic centers [87,88,89]. Moreover, it is not uncommon for spheroids to show a self-renewal and differentiation deficit due to a lack of stem or progenitor cells. These two reasons may partly explain why the culture of spheroids over several months seems challenging. The cell fate may be different depending on the method of spheroid formation, size, or microenvironment.

The hanging drop method can be performed either in specialized plates or, more simply, with a lid of a petri dish [88], which consists of depositing a drop of medium containing the cells on the lid and flipping it over to form the spheroid (Figure 2A) [126]. Specialized plates can be used to facilitate the production of spheroids using the hanging drop method. A small volume of cell solution is deposited in an upper funnel shaped well, and as the cells pass through the micro-well, they aggregate to form a spheroid (Figure 2B) [93].

The advantages of the hanging drop method are in the maintenance of in vivo like cell–cell interactions. These advantages include: the absence of a matrix or scaffold that could potentially interfere with the spheroid formation, the defined and homogeneous size of the spheroids, which require a small number of cells for their formation, and the precise control of spheroid formation in cases involving the co-culture of different cell types [126]. However, changing medium is difficult since the spheroid in formation must not be disturbed. During seeding, only a small volume is needed [126] as an excessive volume would cause the drop to fall by gravity. Lastly, the lid method can be time consuming and requires plate changes to perform the analyses.

The scaffold-based method is often prepared using natural or synthetic hydrogels and can be carried out using two techniques: either the culture plate is first coated with a hydrogel, and the cells are then deposited on the surface and cultured under agitation to promote their adhesion and the formation of spheroids while in contact with the matrix (Figure 2C); or the hydrogel and the cells are deposited simultaneously on the plate to induce spheroid formation within the scaffold (Figure 2D) [94]. The goal of using this method is to reproduce the role of ECM in vivo, and, thus, promote cell–ECM interactions thanks to scaffold facilitating cell adhesion and migration [83]. The main advantage of the scaffold-based method is to be able to use a scaffold with mechanical or structural properties close to those of native tissues [66,94]. The use of synthetic scaffolds can allow better control over the environment, while the use of natural scaffolds is centered on promoting better cell integration. The main drawbacks of this method are the disadvantages related to the nature of the chosen scaffold. For natural scaffolds, these include variability between batches, low mechanical properties, and rapid degradation of the material. Conversely, the main disadvantage for synthetic scaffolds is biocompatibility [85,86].

Spheroids can be fabricated in low-adhesion plates or non-adherent surfaces through cellular assembly [17,34,91]. In low adhesion-plates, wells have a defined geometry (rounded, V-shaped), so that only one spheroid per well is formed with the geometry given by the well [127]. In non-adherent plates, the surface is covered with a neutrally charged hydrophobic coating, which prevent cells from binding to the plate surface, and, in doing so, forces them to bind to each other, causing spheroid formation. The main advantage of using these plates is that all the steps of formation, propagation, and spheroid aggregation can take place in the same plate, in a high throughput manner. However, the main disadvantage of this method is the formation of heterogeneous sized spheroids. Moreover, the preparation of spheroids with few cells is difficult, as well as the adjustment of the adequate cell ratio when a co-culture needs to be performed.

Spheroids can also be formed using suspension culture techniques in spinner flasks or bioreactors [89,95,96]. The principle of these techniques is to promote the formation of spheroids under dynamic culture conditions. The agitation promotes cell–cell interactions (thus spheroid production) while preventing cell adhesion to the flask or bioreactor walls. The main difference between the two systems is the type of rotation used. In spinner flasks, the central stem or agitator of the flasks is in motion, whereas, in bioreactors. the whole system rotates [97]. A suspension culture method provides a substantial production of spheroids and allows for high throughput.

However, it also has several drawbacks which can be a constraint in co-culture situations.

The necessary equipment can be very expensive, and the spheroids produced can have both heterogeneous size but also heterogeneous compositions. Moreover, the stronger shear forces observed in spinner flasks as compared to bioreactors could alter cell physiology [97,98].

Finally, spheroids can be formed by magnetic levitation [99,100]. The cells are treated with a hydrogel containing magnetic beads and then seeded on low-adhesion plates with a magnet in the lid. Attracted by the magnet, cells gather at the air/liquid interface, where they aggregate and form spheroids. The rapidity with which spheroids can form makes this method an advantage. However, magnetic beads are generally expensive and can sometimes be toxic to the cells. Moreover, with this method, the number of cells available for the spheroid formation is limited by the number of beads.

When not combined with other technologies such as microfluidic systems, spheroids are mainly used in the study of cancers.

#### 3.2.3. Organoids

Organoids are an advanced model of spheroids. They are formed from the spontaneous organization of embryonic stem cells, IPSCs, or reprogrammed cells from primary cultures using serum-free cultures [13,35,128,129]. In organoids, stem cells develop over time and tissues organize through self-regulated processes (migration, polarization, and spatio–temporal signals) which lead to the formation of complex structures, such as various regions of the brain [130,131]. Once formed, organoids can vary in size from 0.5 mm to 4 mm and can be grown over a period as long as one year [45,132]. As with spheroids, there are different ways in which organoids can be created. They can be formed by a scaffold-based approach like that of spheroids, where cells are mixed with a scaffold before being deposited as domes in culture plates [131]. Another method is to form embryoid bodies using stem cells. These embryoid bodies are then embedded in Matrigel^TM^ and put into a bioreactor or spinner flasks to form organoids [13,14,128,130]. Finally, the last method consists of forming organoids at the air–liquid interface where stem cells, which can be embedded in Matrigel^TM^, are cultured on a porous membrane in contact with the culture medium [133]. Thanks to their ability to develop in different regions of the body and to preserve the organizational capacities of several tissues, organoids have become one of the best tools to summarize the complex interactions of tissues. They can also be used to recapitulate several diseases that are difficult to model in animals. As a result, organoids are widely used, among others, in the study of genetic diseases, early brain development, neurodevelopmental disorders, or neurodegenerative diseases such as AD, PD, or Huntington’s disease [13,14,35,36,129,132,134]. However, at the time of writing this review, progress is being made with ALS, but there is currently no organoid model for this disease. Duval et al. have been able to develop spinal cord organoids from IPSCs, so it is feasible to consider the same strategy while using IPSCs from ALS patients to better understand the disease [135]. Although organoids are a model that has led to many advances, they have some drawbacks. Due to their spontaneous formation, they can be heterogeneous in size and shape. In addition, organoid culture is difficult, and their reproducibility is rather low due to the lack of standardized protocols [101]. The use of Matrigel, whose composition can vary from one batch to another, can also lead to variations between organoids. Like spheroids, and even more importantly because of their larger size, oxygen and nutrient diffusion may not reach their center, leading to necrosis and detrimental signal secretion [101]. Finally, since organoids may lack some essential non-neuronal cells and are made of IPSCs, it may be difficult to recapitulate age-related diseases. Notably, astrocytes, oligodendrocytes, and microglia often tend to be overlooked in the modeling of neurodegenerative diseases, whereas these cells could have an important role in disease initiation or progression, such as in synaptogenesis, myelination or inflammation [102,136].

#### 3.2.4. 3D Bioprinting

3D bioprinting is a more technological and robotized 3D modeling technique than the methods described above. The concept consists of printing a 3D scaffold while incorporating cells, spheroids, or organoids within to reproduce a 3D tissue or organ [107,108]. The 3D printing is performed using a bioink (which contains a living cell type, a gelatinous biomaterial, and a biomolecule (or a scaffold-free bioink) and is performed layer by layer in an automated way according to a predefined pattern [107,109,137,138]. Three-dimensional bioprinting aims at automatically producing adjustable, but, above all, reproducible 3D models. One advantage of 3D bioprinting is the ability to adjust the size, shape, and porosity of the construct while creating a biocompatible and hydrophilic construct that remains stable in culture, but that also promotes cell growth and communication [139,140]. The key factors in bioprinting are cells, bioink, and biochemical factors. Some details briefly discussed here are well detailed in Bishop’s review [111]. The bioink (often hydrogels) must have capacities that give it not only a mechanical strength role, but also biochemical support. Several properties are often taken into account when choosing the bioink, namely biodegradability, biocompatibility, mechanical properties (viscosity, elasticity), and chemical properties, to name a few [107,140,141]. When designing tissues, the bioink must also allow cells to grow, proliferate, differentiate, and communicate with each other [142]. As previously discussed, the choice of the bioink will depend mainly on the desired characteristics of the tissue which is to be reproduced. Different types of hydrogels are often chosen to use as the bioink when ECM-like characteristics are desired [111,123]. For a more modular side of the physicochemical characteristics, it will be necessary to turn to synthetic scaffolds [107,108,123] whereas natural scaffolds are better for an optimal biocompatibility [140,143,144]. As for the biochemical factors used, their role in cell maturation or differentiation must also be considered.

**Figure 3:**
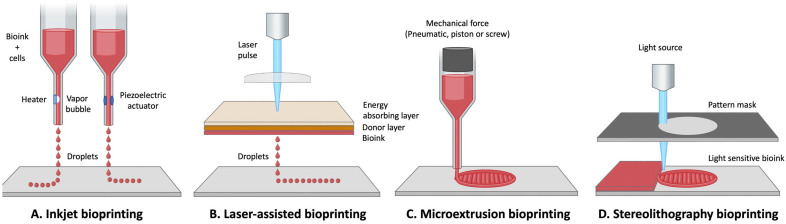
Common bioprinting techniques used to prepare tissues. Inkjet bioprinters controlled by thermal or piezoelectric forces deposit small droplets of bioink and cells to build tissues layer-by-layer (**A**). Laser-assisted bioprinting uses a laser absorbed by an energy absorber layer to heat a donor layer, which forms a bubble propelling the bioink and cells onto the substrate (**B**). Microextrusion bioprinters deposit a cell-laden bioink solution via pneumatic, piston, or screw mechanical force (**C**). Stereolithography bioprinters use UV or visible light to selectively cross-link light-sensitive bioinks layer-by-layer to build a 3D tissue (**D**). Figure produced with Biorender.com.

Several bioprinting techniques are available, each with their own specific advantages and disadvantages (Figure 3). First, the inkjet technique, which is adaptable to both 2D and 3D methods, consists of a one by one non-contact projection of a bioink micro-droplet onto a collection plate, and according to a precise design using thermal, electrical, or piezoelectric forces (Figure 3A) [145,146]. This method is a fast, low-cost technique compared to others described below, and allows cell survival above 85% [112,113,114]. However, inkjet bioprinting is not very precise, and is not suitable for complex tissue reconstruction. Furthermore, it requires a low-viscosity bioink, which is therefore more likely to produce tissues that have low mechanical properties and puts the physical 3D structure at risk of sagging [114,115].

The microextrusion bioprinting method is the most frequently used (Figure 3C). It consists of ejecting the bioink through a micro-nozzle where the flow is regulated using adjustable pneumatic or mechanical forces [112]. The bioink is distributed in a continuous flow following a computer-designed pattern [18,108,138]. Unlike the inkjet method, bioprinting by microextrusion permits the use of bioink with high cell density and high viscosity [112]. With this method it is possible, for example, to dispense spheroids [116]. However, the major disadvantage of this technique is the low cell viability that can result, which can oscillate between 40% and 80% [117]. Indeed, a decrease in cell viability can occur due to the pressure exerted on cells during the extrusion of the bioink through the nozzle [111].

Another bioprinting method uses a laser-assisted process (Figure 3B). In this method, a laser pulsed by the bioprinter, passes through a focusing lens, and is directed onto a printing ribbon that consists of a glass slide coated with an absorbent metal such as gold or titanium, on which a layer of bioink is deposited. When the laser hits the layer with the gold, a high-pressure bubble is created, which then releases and propels a droplet with the cells contained within the bioink towards the collection plate [109,111,112,118,142]. This method is very precise and has a high resolution of up to one cell per droplet [118]. Laser-assisted bioprinting can be used for modeling very complex tissues [142]. Like microextrusion bioprinting, this method allows the use of bioink with high cell density and high viscosity [118]. In addition, there is no nozzle, so obstruction and cell deformation problems are avoided, which keeps cell viability around 95% [109]. Generally, the printing process is very fast, but ribbon manufacturing can be time-consuming. Regrettably, it is a technique that remains very expensive [112].

The last 3D bioprinting method is stereolithography (Figure 3D). This method uses ultraviolet light, but is based on the same principle as laser-assisted bioprinting. A laser is emitted by the printer, reflected by a mirror, and then hits the bioink in a very precise pattern, so that the polymerization is performed layer by layer [107,147]. This method is very fast, has a high degree of precision in the manufacturing process [119] and maintains a good cell viability of about 90% [120]. However, the stereolithography bioprinting method has some drawbacks, such as the use of strong ultraviolet radiation [111,121]. In addition, few materials are compatible with this printing technique, since it requires materials that are both biocompatible and biodegradable [121].

Although 3D bioprinting modeling is widely used for certain tissues such as bone or cartilage reconstruction, its use remains rare for nerve tissues. Creating a bioink for nerve tissues can be difficult because of the fragility of the cells that compose it [110]. Moreover, with this type of modeling, it is possible to be confronted with technical limits, such as the collapse of the construct during layer-by-layer printing [110,111]. However, impressive achievements were realized in the reconstruction of peripheral nerves (for which 3D bioprinting seems particularly well suited) and in spinal cord or brain-like structure development [138,148]. Such constructs made of patient-derived cells could be promising tools to model neurodegenerative diseases.

Yet, due to the high cost of printers and bioinks, 3D modeling techniques do not seem to be ready for large-scale production yet, even if it is currently possible to make heart valves, skin, or ears.

#### 3.2.5. Microfluidic Systems

Microfluidic systems are composed of several chambers connected by microchannels through which fluids flow. Microfluidic chambers are often manufactured by lithography, which allows for high architecture precision, good reproducibility, and excellent optical clarity [74,149,150].

This method has several advantages. First, microfluidic chambers can be adapted to both 2D and 3D cell culture, and can be used for both high and low cell densities [74,149,151]. Moreover, it offers great control over the spatio–temporal environment. It is indeed possible to regulate the microfluidic supply, thus having better control of pH, temperature, oxygen/nutrient supply, or waste removal [43,72,104,150,152]. Sensors, activators, and electrodes can be added to the system, in the case of electrophysiological studies, for example [104,152]. Finally, the cellular response to shear stress, compression, and tension forces can be studied [12,105,153].

Microfluidic chambers are often used to model the brain (especially the blood–brain barrier), to study axonal growth, and neurodegenerative diseases [71,74,149,154,155]. They are also used to study co-culture models, as well as molecular secretions between different culture compartments [92,149]. Even though these culture systems have many advantages, they do not exactly mimic the 3D microenvironment or the physiology of tissues or organs. Moreover, they require specialized equipment (pump, connectors, etc.). In his review, Halldorsson points out several differences, such as different culture surfaces, reduced media volumes, and different cell ratios, as well as specific media change methods [106].

#### 3.2.6. Organs-on-Chips

**Figure 4:**
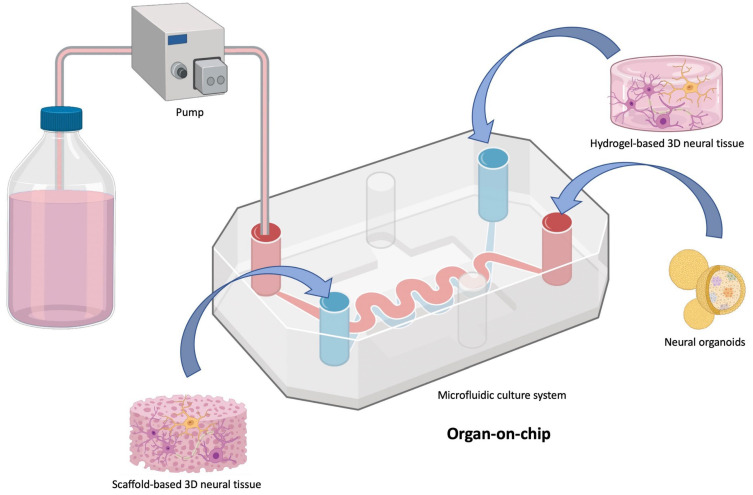
Principle of organ-on-chip development. Various types of 3D tissues (such as scaffold-based, hydrogel-based neural tissues, and neural organoids) can be cultured together in a microfluidic system. The effect of one tissue onto another can be investigated by selective medium flow diffusion. Figure produced with Biorender.com.

Organ-on-a-chip is a tool derived from microfluidic systems (Figure 4). It is a system composed of several single microchambers, or multiple microchambers each connected by microchannels [71]. Organ-on-a-chip is a microfluidic system allowing reproduction of human physiology on a small scale. The goal is not necessarily to recreate an organ in its entirety, but rather to recreate a functional unit of this organ, which is characteristic of one of its functions [12,16,72,102]. The concept is the same as the one described above for microfluidic chambers. Cells are seeded in the scaffold (often a hydrogel), which gives them their 3D environment and promotes cell survival, proliferation, and differentiation as described above [81,82,83]. It is possible to play with the scaffold composition (as well as its stiffness) and to integrate (or not) a chemical gradient.

This 3D system could replace animal use for some studies. In short, the costs to use organs-on-a-chip are probably lower than those generated by animal purchase and housing. Moreover, with this system, there is no difficulty in isolating a specific organ (or a functional unit), which is in contrast to animal use. Using patient derived-cells in conjunction with organ-on-a-chip could also avoid the use of transgenic animals, along with the uncertainty from drawing parallels between animal use and humans. The advantages of this tool are similar to those listed for microfluidic chambers: i.e., excellent control of the chemical, physical or mechanical microenvironment; and good control of seeding and cell arrangement [155,156]. Organs-on-chips are more elaborate systems than 2D or 3D simple microfluidic chambers. They allow, for example, to study cell–cell or cell–ECM interactions, or to study the functional unit response to stimuli (drugs, pathogens, etc.) [157,158]. Thanks to the combination of computerized 3D bioprinting using microfluidic systems designed with the incorporation of living cells, organs-on-chips can allow structural resemblance at both physiological and pathophysiological levels [12,159,160,161]. The system can also be automated, requires a very low amount of culture medium, and can be designed to image cells in real time by confocal microscopy. Last but not least, it is possible to grow spheroids or organoids in these systems without a hydrogel, and this could allow for toxicity studies or drug testing [162,163].

However, organs-on-chips are a more complex and more computerized system than simple 2D or 3D microfluidic chambers, but they are more challenging to use, and the slightest technical problem can lead to an interruption of the experiment.

Organ-on-chips are widely used in neuroscience and neurodegenerative diseases, such as PD, AD, or ALS, because they allow for the study of very elaborate and complex structures [16,160,164,165].

## 4. Conclusions

Animal models are still widely used in research, especially in pre-clinical studies. In vivo models have allowed for a great advance in knowledge, but their potential remains limited. The parallel between humans and animals is not always as obvious as one might think, and it is often difficult to model a pathological phenomenon in its globality when taking into account all the associated epigenetic characteristics.

In vitro 2D or 3D cell culture allows for the reduction of animal use. Cell culture has the advantage of being able to study cellular mechanisms more independently than in an in vivo model. Two dimensional cell culture is more simplified than 3D, and has proven to be very useful for studying cell interaction or cell behavior, in response to various stimuli. However, cells that are grown in monolayers are exposed to large amounts of nutrients, oxygen, and growth factors, which give them an unnatural phenotype. In addition, the lack of ECM and 3D structural complexity in the physiological environment is a major disadvantage. With the advancement of technologies, a more sophisticated 3D cell culture has come to remedy certain shortcomings. Scaffold-based techniques allow the integration of a chemically, physically, and mechanically malleable ECM, which favors cell–ECM interactions. Moreover, cell seeding is manageable, and 3D culture allows for the combination of multiple cell types, which can create a tissue-like microenvironment when these cells are placed in close proximity to each other. More complex spheroid and organoid settings allow cell self-organization, as well as physiologically optimal cell interactions. However, culture medium circulation often remains heterogeneous, and the structure centers are often hypoxic. The combination of these models with more complex 3D bioprinted tissues, microfluidic systems, or organs-on-chips (all produced under automated processing), offers the possibility of integrating controllable fluid flows, tension, and compression forces (as well as a very high precision) into the model architecture and cell seeding.

At this time, there is no particular model which is able to recreate all the complexity found in neurodegenerative diseases, but it is certainly all the advances with 2D and 3D in vitro models (as well as the combination of several 3D in vitro models such as organs-on-chips) that will allow very complex and elaborate platforms to be achieved.

## Figures and Tables

**Table 1:** Summary of the 4 main neurodegenerative diseases with their main symptoms, causes, and biological hallmarks.

Disease	Symptoms	Cause	Biological Hallmarks
**AD** Alzheimer disease	Cognitive impairment	Unclear, multifactorial	Neuronal degeneration, β-amyloid plaques, neurofibrillary tangles, hyperphosphorylated tau, Neuroinflammation
**PD** Parkinson disease	Motor impairment	Unclear, multifactorial	Dopaminergic neuron degeneration, α-synuclein aggregates, Lewy bodies
**HD** Huntington disease	Cognitive and motor impairment	Autosomal dominant disorder, CAG expansion in the huntingtin gene	Neuronal degeneration
**ALS** Amyotrophic lateral sclerosis	Motor impairment, frontotemporal dementia	Unclear, multifactorial, 10% of patients with various mutations (SOD1, FUS, TARDP, etc.) or DNA repeat expansion (C9orf72)	Motor neuron degeneration, neuroinflammation, ubiquitin inclusions, misfolded SOD1 accumulation

**Table 2:** Overview of in vitro 3D models.

Methods	Size	Culture Time	Advantages	Disadvantages	Ref
**Scaffold-based approach**	Scaffold dependent, up to a few mm	Scaffold degradation dependent (1 day to several months)	Cell adhesion, proliferation, differentiation depending on scaffold physicochemical properties Oxygen and nutrients transport depending on scaffold permeability High reproducibility ECM organization control	Cellular adhesion and growth changes depending on scaffold material Cell behavior changes depending on cellular topography distribution	[6,45,46,81,82,83,84,85,86]
**Spheroids**	<1 mm	Up to 2 months	Reproducible Nutrients and oxygen gradients Optimal cell–cell and cell–ECM interactions Long term culture (≈2 months)	Simplified architecture Hypoxic center Self-renewal deficit	[45,87,88,89]
**Low-adhesion plate/** **Non-adherent surface**			Easy to use All steps in the same plate High throughput	Heterogeneous size Find an appropriated cell ratio for co-culture Spheroid formation with few cells	[34,90,91,92]
**Hanging drop**			No scaffold needed Homogeneous size Low number of cells required Co-culture control	Low throughput Need to change plates for assays Time consuming Challenging media renewal methods Difficult long term culture	[86,89]
**Hanging drop plate**			High throughput Homogeneous size Low number of cells required Co-culture control	Challenging media renewal methods	[88,90,93]
**Scaffold-based method**			Mimic in vivo environment Cell–ECM interactions High throughput	Scaffold changes between batches Natural hydrogels: weak mechanical properties, rapid degradation Synthetic scaffold: biocompatibility issues	[66,85,86,94]
**Suspension cultures: spinner flasks/bioreactor**			Cell–cell interactions High throughput Mass production	Specialized equipment High shear forces (bioreactor < spinner flask) Heterogeneous size and composition	[90,95,96,97,98]
**Magnetic levitation**			Fast spheroid formation ECM intrinsic formation	Expensive beads Limited cell number	[90,99,100]
**Organoids**	0.5–4 mm	Up to 1 year	Long term culture (≈1 year) Spontaneous formation Mimics embryonic development Cell self-organization Complex tissue organizational capacities like in vivo	Heterogeneous shape and size Low reproducibility Hypoxic centers Lack of key cell types (most of time) Instability between batches	[45,101,102,103]
**Microfluidic System**	<1 mm	Scaffold degradation dependent (1 day to several months)	Spatio–temporal environment control Dynamic culture Co-culture Cell-patterning control	Challenging technical side (microcircuits) Specialized equipment (pump, device) Challenging media renewal methods Different culture surfaces	[45,72,92,104,105,106]
**3D Bioprinting**	Scaffold dependent, up to a few cm	Scaffold degradation dependent (1 day to several months)	Robotized Cell-patterning control ECM configuration control Possible combination of 3D models	Expensive (bioink, printers) Possible collapse of layers	[45,107,108,109,110]
**Inkjet**			High speed Low cost >85% cell viability	Low precision Few cells, low viscosity	[111,112,113,114,115]
**Microextrusion**			Easy to use High cell density and high viscosity bioink	Low cell survival: 40% viability Cell structure distortion	[18,111,116,117]
**Laser-assisted**			High cell density and high viscosity bioink High precision 95% cell survival	High cost Time-consuming ribbon manufacturing	[109,111,118]
**Stereo-lithography**			High precision >90% cell viability	Strong UV light use Polymer biocompatibility and biodegradability	[111,119,120,121]

## Data Availability

Not applicable.
